# Circulating serotypes of dengue virus and their incursion into non-endemic areas of Pakistan; a serious threat

**DOI:** 10.1186/s12985-016-0603-6

**Published:** 2016-08-26

**Authors:** Amjad Ali, Habib Ahmad, Muhammad Idrees, Fazli Zahir, Ijaz Ali

**Affiliations:** 1 Centre for Applied Molecular Biology, University of the Punjab, Lahore, Lahore-53700 Pakistan; 2 Department of Genetics, Hazara University Mansehra, Mansehra, Khyber Pukhtunkhwa Pakistan; 3 Vice Chancellor, Hazara University Mansehra, Mansehra, Pakistan; 4 IBGE, The University of Agriculture, Peshawar, Khyber Pukhtunkhwa Pakistan; 5 Department of Biosciences, COMSATS Institute of Information Technology, Islamabad, Pakistan

**Keywords:** Circulating serotypes of dengue virus, Dengue infection, Epidemiology of dengue, Swat, Dengue Pakistan, Khyber Pakhtunkhwa

## Abstract

**Background:**

Dengue virus is circulating in Pakistan since 1994, which causes major and minor outbreaks in many areas of the country. The incidence of dengue in Pakistan in past years mainly restricted to parts of Sindh and Punjab provinces. As such, a severe dengue outbreak appeared in Pakistan in 2011, particularly in Punjab province with Lahore as the most hit city (290 deaths). In 2013, for the first time in the history of Pakistan, dengue outbreak erupted in Swat District, Khyber Pakhtunkhwa, which claimed more than 57 lives. Hence this study was conducted to document circulating serotypes of dengue virus in Pakistan in 2011 and 2013 dengue outbreaks in two different territories/areas of the country.

**Methods:**

In total, 1340 blood samples from people having dengue (ELISA positive) and/or dengue like symptoms from various cities/areas of Punjab and Swat, Khyber Pakhtunkhwa (KP) were collected and analyzed by reverse transcription polymerase chain reaction (RT-PCR) using serotype specific primers.

**Results:**

The results indicated that all the four dengue virus serotypes were circulating in Punjab Province with highest frequency of DENV-2 (41.64 %) and DENV-3 (41.05 %). Similarly, DENV-2 (41.66 %) and DENV-3 (35.0 %) were dominant serotypes detected in KP-based people lived in Punjab. On the other hand only DENV-2 (40.0 %) and DENV-3 (60.0 %) were detected in Swat District. Furthermore an important observation noted in this study was mixed infection of DENV-2 and DENV-3 in Punjab in 2011 (3.81 %) and in people from KP infected in Punjab (8.33 %) which may account for the high mortality and morbidity rates as compared to previous outbreaks. Over all male population was mostly infected as compared to females and people in the age group between 15 to 45 was the highest infected group.

**Conclusions:**

The findings of this study indicate that all four serotypes of dengue virus are circulating in Punjab whereas serotypes 2 and 3 introduced for the first time into Swat, KP in 2013; about 600 km away from Lahore, Punjab. Overall dengue virus serotypes 2 and 3 were the major outbreak-causing serotypes in Pakistan in 2011 and 2013. Dengue outbreak in Swat may be the continuation of previous dengue outbreaks in Punjab but it needs further research and investigation.

## Background

Dengue fever is an important mosquito-borne viral disease, caused by one of the four closely related but antigenically distinct dengue virus serotypes (DENV-1 to 4). According to World Health Organization (WHO), the annual incidence of dengue infection is 100 million, of which approximately 500,000 patients develop dengue hemorrhagic fever (DHF) that may lead to dengue shock syndrome (DSS) with a mortality rate of more than 2.5 % [1–3]. DENV has a positive sense RNA genome (genus *Flavivirus*; family *Flaviviridae*) of approximately 11 Kb in size. Infection with one serotype does not provide immunity against other serotypes, so a person living in an endemic area could have up to four DENV infections during his/her life span [4, 5]. All four serotypes of dengue are able to produce DHF or DSS in humans after infection is established [6]. In the tropical and subtropical climates of the world, the incidence of dengue is continuously on the rise due to suitable climatic conditions for dengue vector growth [7]. Furthermore, antibodies raised against the membrane protein prM are known not to neutralize the virus but they can cause the antibody-dependent enhancement (ADE) [8–10]. Due to ADE, and in order to protect individuals from DENV infections, it becomes critical to survey the dengue serotypes in any particular area.

In August 1994, the first confirmed outbreak of DENV was reported in Karachi, Pakistan. Since then, moderate to severe dengue outbreaks were observed in many areas of Pakistan. Dengue infection did not contain to southern Pakistan (Karachi) but transmitted to northern parts (Punjab) of the country with the passage of time that caused great losses in terms of mortalities and morbidities [11–15].

In 2011, a devastating dengue outbreak reported in Lahore, the capital city of the Punjab, Pakistan. According to Health Departments in Pakistan, more than 23,000 people were hospitalized, of which at least 365 people died due to DHF and DSS [13, 14]. DENV-2 and DENV-3 and a single case of DENV-4 were previously reported the 2011 dengue outbreak in Punjab [16].

Unprecedentedly, just 1 year after the major outbreak in Lahore, a sever dengue outbreak occurred in 2013 in Swat District, Khyber Pukhtunkhwa (KP), which is about 600 km away from Lahore and has relatively temperate climatic conditions (average annual rainfall exceeds 1000 mm and mean annual temperature of about 18 °C) in contrast to Lahore, Punjab (average annual rainfall recorded is 430–767 mm and mean annual maximum temperature of about 28–31 °C) [17, 18].

The outbreak in Swat claimed 57 lives and thousands of people were hospitalized. Therefore, this study was conducted to determine the exact distribution of DENV serotypes in the 2011 and 2013 outbreaks and to shed light on the overall situation.

## Methods

### Collection of samples from patients during the years 2011 and 2013

During the 2011 dengue outbreak in Punjab, blood samples (*n* = 600) were collected from Enzyme-linked immunosorbent assay (ELISA) positive hospitalized patients (461 patients from five districts in Punjab province and 139 patients from six districts of KP-based people, lived in Punjab) (Table 1). ELISA test for the initial diagnosis of DENV had conducted by the respective health centers and then we collected blood samples from those patients (ELIZA positive) for further analysis such as RT-PCR. Similarly, during the 2013 outbreak of DENV in Swat District, blood samples (*n* = 740) were randomly collected from the hospitalized patients at Saidu Medical College Hospital in Swat, experiencing dengue like symptoms (Table 1). Blood samples were shifted to the Institute of Biotechnology and Genetic Engineering (IBGE), The University of Agriculture, Peshawar, Pakistan. Blood samples collected in Swat, KP were analyzed initially by SD *Dengue* Duo strips (Standard Diagnostics, Korea) tests for IgM, IgG and DENV NS1 glycoproteins. Statistical analysis was done using SPSS version 20.

**Table 1:** Collection of samples during 2011 and 2013 dengue outbreaks and distribution of DEVN serotypes in affected people

Province/Year	District	Samples	PCR + ve	DENV1	DENV2	DENV3	DENV4	Mixed	M/F	Age group [Years]		
										<15	15–45	>45
Punjab/2011	Lahore	322	282	11	121	115	25	10	154/128	57	164	61
	Sargodha	58	28	3	9	8	5	3	17/11	5	16	7
	Sheikhupora	41	16	0	7	8	1	0	9/7	3	9	4
	Kasur	29	13	0	4	8	1	0	9/4	2	7	4
	RYK	11	2	0	1	1	0	0	2/0	0	2	0
	Total	461	341	14 (4.10 %)	142 (41.64 %)	140 (41.05)	32 (9.38 %)	13 (3.81 %)	191/150	67 (19.64 %)	198 (58.06 %)	76 (22.28 %)
KP/2011	Peshawar	63	26	3	10	8	2	3	21/5	4	15	7
	Mardan	25	11	1	5	3	1	1	8/3	1	8	2
	Nowshehra	19	8	1	4	2	0	1	6/2	1	4	3
	Kohat	6	2	0	0	2	0	0	2/0	0	2	0
	Charsadda	10	5	1	2	2	0	0	4/1	1	3	1
	Swat	16	8	0	4	4	0	0	7/1	1	6	1
	Total	139	60	6 (10.0 %)	25 (41.66 %)	21 (35.0 %)	3 (5.0 %)	5 (8.33 %)	48/12	8 (13.33 %)	38 (63.33 %)	14 (23.33 %)
KP/2013	Swat	740	200	0	80 (40.0 %)	120 (60.0 %)	0	0	159/41	42 (21.0 %)	89 (44.50 %)	69 (34.50 %)

*KP* Khyber Pakhtunkhwa, *RYK* Rahim Yar Khan, *M/F* Male/Female, *DENV* Dengue Virus

**Fig. 1:**
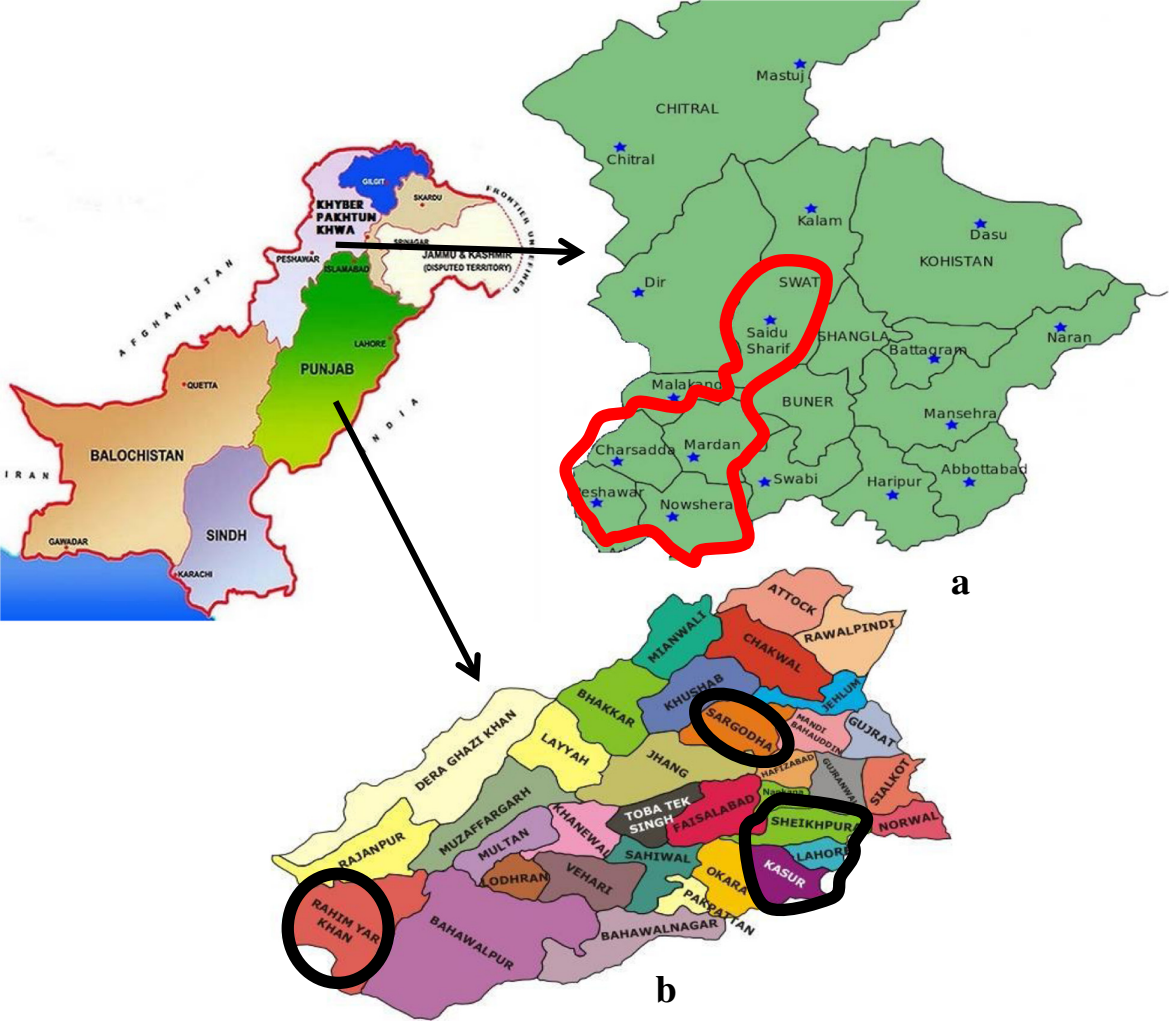
Provinces and Districts hit by dengue virus in 2011 and 2013 in Pakistan. **a** Map of Khyber Pakhtunkhwa, DENV hit areas are encircled. **b** Map of Punjab, DENV hit districts/areas are encircled

Information about the patients and/or samples collected from various geographical areas of Pakistan in both these two dengue outbreaks is provided in Fig 1.

### RNA extraction and serotype-specific PCR

RNA from all serologically positive blood samples was extracted using QIAGEN QIAamp viral RNA mini kit (QIAGEN, Hilden, Germany) according to the manufacturer’s instructions. The extracted RNA was used for the identification of the DENV serotypes (1–4) using type-specific reverse transcription-polymerase chain reaction (RT-PCR) as described previously [1, 19].

## Results

**Fig. 2:**
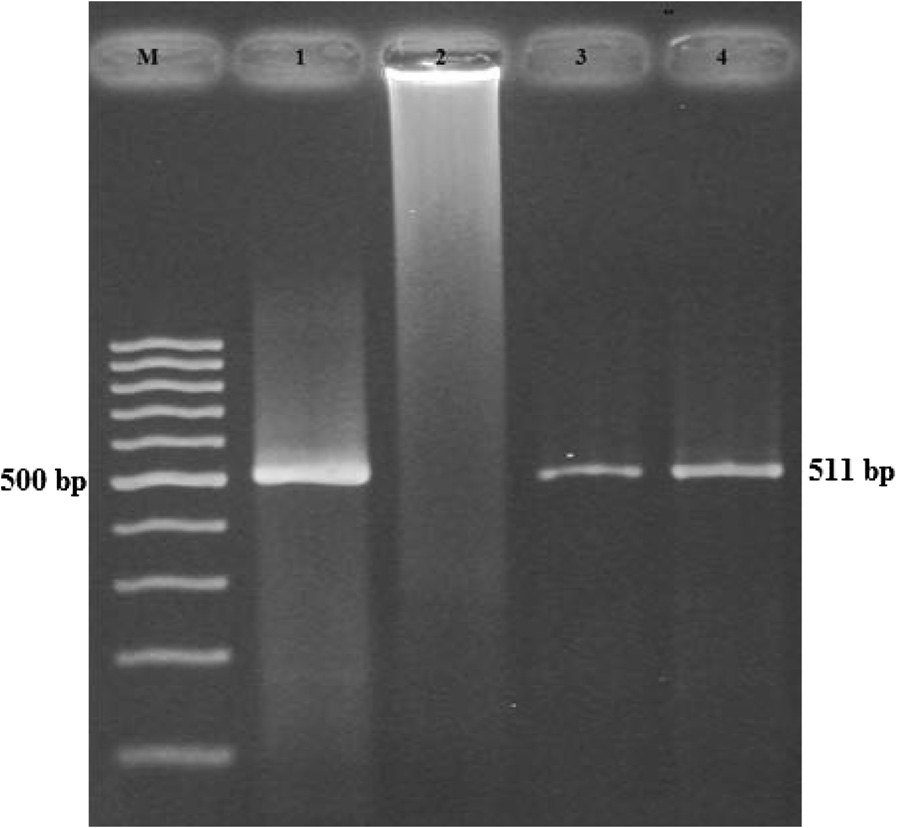
Gel photograph of the first round RT-PCR product. M: 100 bp DNA ladder (GeneRuler); 1: Positive control (provided by Molecular genetics lab in IBGE, The University of Agriculture Peshawar, Pakistan); 2: Negative control; 3–4: 511 bp specific bands of the first round RT-PCR reaction, representing DENV presence in serum samples of patients

Results of this study indicated that all four serotypes of DENV (1–4) are circulating in Punjab province and DENV-2 and DENV-3 in Swat, KP. DENV specific bands (511 bp) of the first round RT-PCR assay along with positive and negative controls are given in Fig 2.

### The 2011 outbreak in Punjab province

**Fig. 3:**
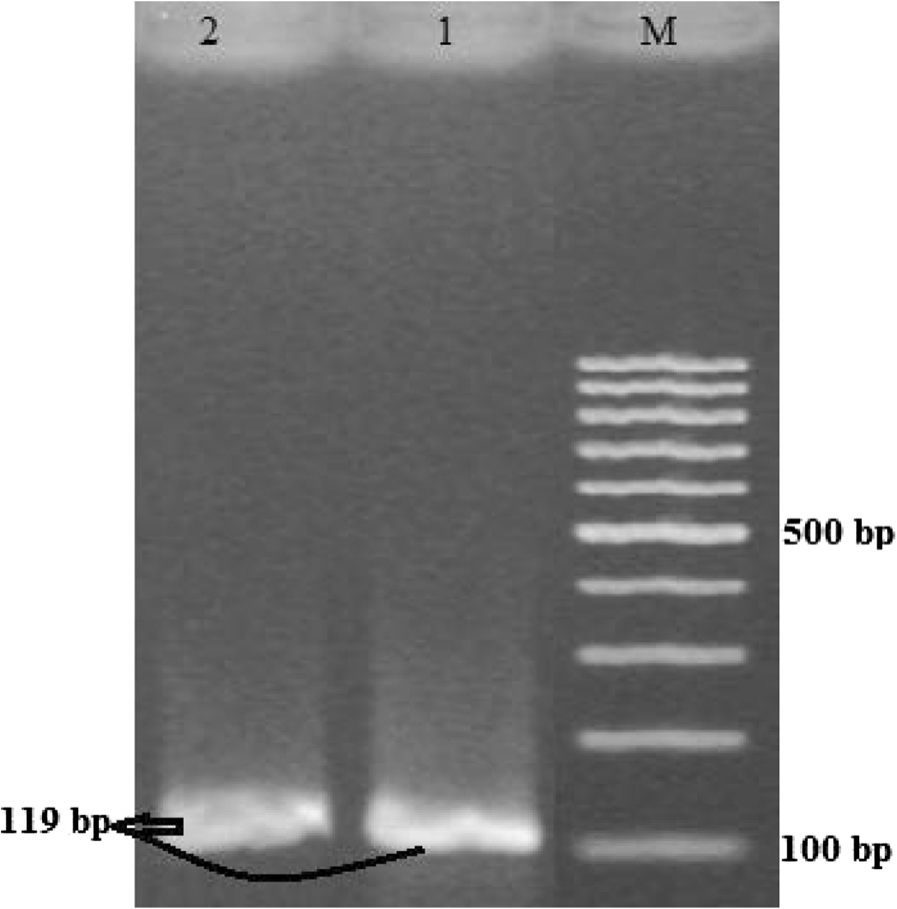
Gel photograph of sample no. 2x, representing DENV-2. M: 100 bp DNA ladder (GeneRuler); 1–2: DENV-2 specific bands of 119 bp size (Sample no. 2×)

**Fig. 4:**
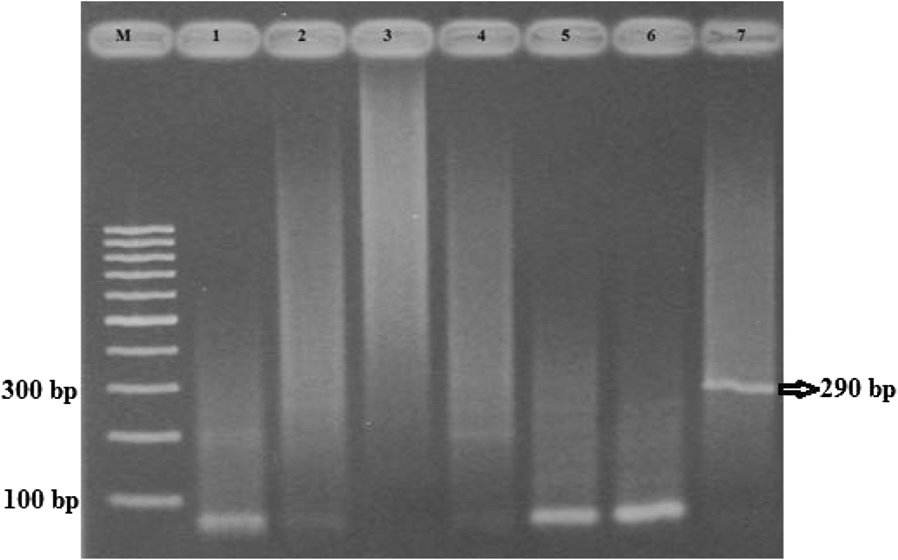
Gel photograph of samples tested for the presence of DENV-3. M: 100 bp DNA ladder (GeneRuler); 1: Sample no. 2z; 2: Sample no. 2y; 3: Sample no. 2w; 4: Sample no. 2v; 5: Sample no. 2u; 6: Sample no. 2 t; 7: Sample no. 2x, showing DENV-3 specific band of 290 bp

In Punjab province, 74 % samples from patients belonged to Punjab (341 out of 461) were positive for DENV-RNA as indicated by RT-PCR analysis and confirmed to have active dengue infection that comprised 56 % (191 out of 341) males and 44 % (150 out of 341) female patients (Table 1). Majority of the actively infected people were from Lahore (83 %) followed by other districts like Sargodha (9 %), Sheikhupura (5 %), Kasur (4 %) and Rahim Yar Khan (0.5 %) (Table 1). Importantly, mixed infections with DENV-2 and DENV-3 in same serum samples were detected in this study; like sample no. 2x that revealed both DENV-2 and DENV-3 (Figs. 3 and 4). Mixed infection comprised of DENV-2 and DENV-3 in patients belonged to Punjab was 3.81 % (13 out of 341). The most dominant DENV serotypes in Punjab province were DENV-2 (41.64 %) and DENV-3 (41.05 %). The incidence of DENV-4 and DENV-1 recorded was 9 and 4 % respectively.

Sample no. 2x along with other samples was tested with specific primers for the detection of DENV-3. Out of the seven samples tested, DENV-3 was detected in sample no. 2x that confirmed the mixed infection (Fig. 4).

Similarly active dengue infection rate as revealed by RT-PCR assay in KP-based patients, lived in Punjab was 43 % (60 out of 139). Male population was mostly infected (80 %) as compared to female population (20 %). Majority of KP-based people who infected in Punjab were from Peshawar (43 %), Mardan (18 %), Nowshehra (13 %), Swat (13 %), Charsadda (8 %) and Kohat (3 %) (Table 1). Mixed infection (8.33 %) of DENV-2 and DENV-3 was also recorded in some patients (Table 1). Individuals between the ages of 15 to 45 years were mostly infected in the 2011 dengue outbreak.

### The 2013 outbreak in Swat, KP province

Analysis of the 740 blood samples collected from patients in Swat District during the 2013 dengue outbreak showed that 618 patients were positive for dengue antibodies. However, active dengue infection was detected in only 200 patients out of 618 as revealed by RT-PCR assay. Only DENV-2 (40.0 %) and DENV-3 (60.0 %) were detected in patients and no evidence of DENV-1 and DENV-2 was recorded in Swat (Table 1).

**Fig. 5:**
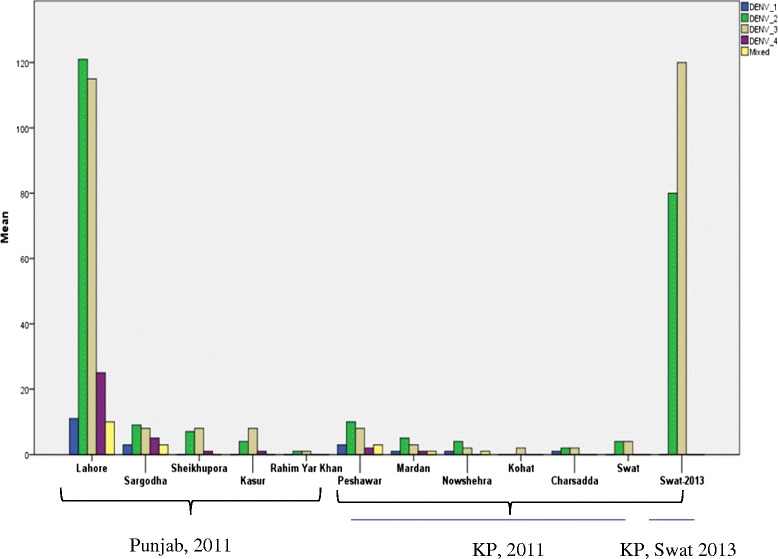
District wise frequency distribution of DENV serotypes in Punjab and KP in 2011 and in Swat, KP in 2013. DENV-2 and DENV-3 are the major outbreak causing serotypes in both the outbreaks as indicated. *Blue*, *green*, *gray*, *violet* and *yellow* colors indicate DENV-1, DENV-2, DENV-3, DENV-4 and mixed infection, respectively

Figure 5 indicates the overall summary of the situation of DENV serotypes, circulating in Pakistan. Frequency distribution of serotypes clearly indicates that DENV-2 and DENV-3 are the dominant serotypes in Pakistan with some mixed infection (Fig. 5).

## Discussion

Dengue has been one of the major causes of hospitalization among people since the 1990s, particularly in South East Asia [20]. Increased population movement and spread of competent mosquito vectors are the factors contributing to the spread and establishment of the disease in temperate areas of the world [21]. Countries bordering Pakistan like India is facing dengue since 1996 and all four serotypes have been documented in Delhi [22]. Similarly, DENV-2, DENV-3 and DENV-4 have been detected in a study conducted in Bangladesh [23]. Prevalence of dengue infection has also reported from Iran [24].

In Pakistan, Dengue virus infection initially started in Karachi in 1994 and gradually expanded to other areas of the country with the passage of time [13, 14]. The geographical location of Pakistan with hot and humid summers particularly in Punjab, is an ideal place for the breeding of the *Aedes* mosquitoes [13] which are responsible for spreading the virus from one location to another. Environmental fragmentation which resulted from severe flood in 2010 further encouraged the breeding of the *Aedes* mosquitoes in Pakistan [25, 26]. Consequently, DENV infection reached epidemic level in 2011 in Pakistan. In this study, patients (*n* = 1340) from 12 different districts of Pakistan were analyzed for prevalence, distribution, and frequency of dengue virus. The results indicated that all four DENV serotypes circulate in Punjab Province and only DENV-2 and DENV-3 for the first time introduced into Swat District, KP (Table 1).

This study showed that during the 2011 dengue outbreak in Punjab, all four serotypes of DENV were prevalent in both genders and capable of active infection in population but DENV-2 and DENV-3 were the most dominant serotypes. Consequently, this type of studies presented here become crucial to determine the possibility of antibody-dependent enhancement (ADE), which usually leads to DSS [2, 3]. Mixed infection in 2011 dengue outbreak highly suggests secondary infection and hence more infectivity and deaths (Table 1).

In 2013 dengue outbreak in Swat, DENV-2 (40 %) and DENV-3 (60 %) were the dominant serotypes isolated from the infected people while no evidence of DENV-1 or DENV-4 was found (Table 1). Another aspect which may account for the presence of higher incidence of DENV-3 in Swat than DENV-2 (unlike in Punjab) may be the role of Ae. Albopictus (Fig. 5). This species is generally related to temperate type of environment in the ruler areas [27] like Swat, but it needs further investigation and we would address it in our coming project.

In Swat, extensive deforestation and a new culture of cultivating orchards; which is currently the major means of income of the majority of people, has brought substantial changes in the agriculture landscaping. Severe flooding in 2010 and the recent DENV outbreak is an indication of the climate change in this scenic valley because dengue incidence is associated with change in climate [28]. Lake of fresh water supply system is another problem afflicting the population now-a-days and that’s why water pools have been dug inside orchards where people keep water for several days which provides enough time and favorable conditions for the breeding of *Aedes* mosquitoes.

Results of this study indicate that the active and juvenile group (15–45 years) was the most affected. Number of the elderly infected people in Swat was considerably higher than those infected during the 2011 outbreak in Punjab. One possible reason may be the healthy environment of Swat District; allows people to remain active and contribute to the working class even in their late sixties and early seventies. More exposure and more activity during the day time provide more chances for the vector bites and hence higher infection rate.

The gender wise distribution of DENV infection indicates no significant difference between the male and female populations (191 & 150 respectively) infected in Punjab during the 2011 outbreak. However, the gender wise difference (48 males & 12 females) in Swat District was quite evident during 2013 dengue outbreak (Table 1). The possible reason for this may be different social set ups at two distant locations with different cultural characteristics. Women are mostly restricted to remain inside homes in Swat District while in contrast there is a huge class of working women in Punjab which increases the chances of contracting the infection.

A considerable number of patients were found to have mixed infection (Table 1) which indicates that the individuals with mixed infection had acquired infection in highly endemic pockets of the cities due to increased breading activity of the mosquitoes during the same season.

The incidence of concurrent or mixed infection in Punjab in 2011 can be attributed to the long history of DENV in the region. In 2006, the first confirmed DENV outbreak erupted in Punjab and since then it is causing sporadic infection and/or major outbreaks. In 2006, 3000 confirmed cases were registered in Pakistan, out of which 52 people died [29]. In 2008, dengue infection continued in Pakistan with 1450 people infected in Punjab, of which 20 died. In that outbreak, ten patients out of 17 in Lahore were positive for DENV-4, five for DENV-2 and two for DENV-3 when analyzed through real time PCR [30]. In 2010, dengue outbreak occurred in Pakistan and more than 4000 cases were reported from Punjab that caused three deaths [13].

DENV outbreak in Pakistan in 2011 was the most severe in the history; affected more than 22,562 individuals in total and claimed 363 lives across Pakistan according to the reports of health departments. According to independent surveys, the number of infected people was more than 35,000 and number of deaths it caused was more than 420. Punjab once again was the most hit Province by DENV that infected more than 21,300 individuals, out of which 337 people died. Though the entire Punjab was affected but Lahore was the most hit city with 17,493 people infected, of which 290 people died. As compared to previous dengue outbreaks in Punjab, mortality and morbidity rate was highest in 2011. The reasons for such high rate of infectivity and deaths as compared to previous infections in Pakistan, may be the long history of dengue in the region, prevalence of all four serotypes (Table 1), mixed infection (Figs. 3 and 4) that usually leads to DHF and DSS [8–10] and climatic suitability for vector species.

This is the first study in Pakistan that documents concurrent infection in 2011 dengue outbreak in Punjab (Table 1). Concurrent infection was not observed in 2013 dengue outbreak in Swat. The possible reasons behind this may be that this was the first incidence of dengue with no previous record of dengue in Swat. Secondly, the infection was in progress in Swat when we conducted this study and ultimately it is clear from the above history that for concurrent infection to appears, co-circulation of multiple DENV serotypes for fairly long time in an endemic area may be required. The incidence of concurrent infections has already been reported in some parts of the world like in Puerto Rico in 1982 [31], in India with overall 66.7 % concurrent infection of DENV-2 and DENV-3 [32] and in Bangladesh, where two patients were co-infected with DENV-2 and DENV-3 while one patients was co-infected with DENV-3 and DENV-4 [23].

We suggest that effective mosquito eradication strategies should be adopted as future outbreaks may cause high mortality due to more chances of secondary infections. Moreover, dengue occurrence in areas like Swat is lethal because Swat is famous for tourism and if dengue persists it would greatly destroy not only tourism in Swat but in the adjacent areas too. Further, thorough genetic characterization of the DENV serotypes should be carried out in order to facilitate futuristic vaccine and/or drug development strategies.

## Conclusions

Dengue virus is continuously affecting the people of Pakistan. Dengue virus is spreading to remote, rural and previously non-endemic areas of Pakistan with DENV-2 and DENV-3 as the major spreading viruses. Rural areas of Pakistan are less developed and lack basic health infrastructure, education and jobs opportunities. Therefore concerted efforts should be employed to check the spread of dengue into theses less developed areas in order to prevent further damage and to protect lives of the common and poor people in particular. Furthermore, research should be initiated to uncover the factors behind this expansion of dengue virus into previously non-endemic areas so that preventive measures may be taken.

## Abbreviations

DENV: Dengue virus
DHF: Dengue hemorrhagic fever
DSS: Dengue shock syndrome
ELISA: Enzyme-linked immunosorbent assay
IBGE: Institute of Biotechnology and Genetic Engineering
KP: Khyber Pakhtunkhwa
RT-PCR: Reverse transcriptase polymerase chain reaction

## References

[1] Ali A, Nasim Z, Rehman R, Farzana AS, Ali S, Zahir F, Iqbal A, Ali I, Khan AW (2013). Dengue virus serotype 2 and 3 causing high morbidity and mortality in Swat, Pakistan. Biohelikon: Immun Dis.

[2] World Health Organization: Dengue hemorrhagic fever: diagnosis, treatment and control. 2nd edition. Geneva: World Health Organization; 1997. http://www.who.int/csr/resources/publications/dengue/Denguepublication/en/. Accessed 2 Dec 2015.

[3] Simmons CP, Farrar JJ, Nguyen V, Wills B. Dengue. N Engl J Med. 2012. doi:10.1056/NEJMra1110265. 10.1056/NEJMra1110265 22494122

[4] Gubbler DJ (1998). Dengue and dengue hemorrhagic fever. Clin Microbiol.

[5] Guzman MG, Halstead SB, Artsob H, Buchy P, Farrar J, Gubler DJ (2010). Dengue: a continuing global threat. Nat Rev Microbiol.

[6] Kyle JL, Harris E. Global spread and persistence of *dengue*. Annu Rev Microbiol. 2008. doi:10.1146/annurev.micro.62.081307.163005. 10.1146/annurev.micro.62.081307.163005 18429680

[7] World Health Organization. Dengue: guidelines for diagnosis, treatment, prevention and control. WHO Press; 2009. http://www.who.int/rpc/guidelines/9789241547871/en/. Accessed 2 Jan 2016. 23762963

[8] Dejnirattisai W, Jumnainsong A, Onsirisakul N, Fitton P, Vasanawathana S, Limpitikul W, et al. Cross-Reacting Antibodies Enhance Dengue Virus Infection in Humans. Science. 2010. doi:10.1126/science.1185181. 10.1126/science.1185181 PMC3837288 20448183

[9] Kliks SC, Nisalak A, Brandt WE, Wahl L, Burke DS (1989). Antibody-dependent enhancement of dengue virus growth in human monocytes as a risk factor for dengue hemorrhagic fever. Am J Trop Med Hyg.

[10] Morens DM, Halstead SB (1990). Measurement of antibody-dependent infection enhancement of four *dengue* virus serotypes by monoclonal and polyclonal antibodies. J Gen Virol.

[11] Chan YC, Salahuddin NI, Khan J, Tan HC, Seah CL, Li J (1995). Dengue hemorrhagic fever outbreak in Karachi, Pakistan. Trans R Soc Trop Med Hyg.

[12] Koo C, Nasir A, Hapuarachchi HC, Lee KS, Hasan Z, Ng LC, Khan E. Evolution and heterogeneity of multiple serotypes of Dengue virus in Pakistan, 2006–2011. Virol J. 2013. doi:10.1186/1743-422X-10-275. 10.1186/1743-422X-10-275 PMC3844417 24007412

[13] Rasheed SB, Butlin RK, Boots M. A review of dengue as an emerging disease in Pakistan. Public Health. 2013. doi:10.1016/j.puhe.2012.09.006. 10.1016/j.puhe.2012.09.006 23219263

[14] Shakoora MT, Ayubb S, Ayub Z (2012). Dengue fever: Pakistan’s worst nightmare. WHO South-East Asia J Public Health.

[15] Jamil B, Hasan R, Zafar A, Bewley K, Chamberlain J, Mioulet V (2007). Dengue virus serotype 3, Karachi, Pakistan. Emerg Infect Dis.

[16] Fatima ZA, Afzal SA, Idrees M, Rafique S, Akram M, Khubaib B, et al. Change in demographic pattern of dengue virus infection: evidence from 2011 dengue outbreak in Punjab, Pakistan. Public Health. 2013. doi:10.1016/j.puhe.2013.03.003. 10.1016/j.puhe.2013.03.003 23973044

[17] National Rural Support Program. Evaluation & Research Report NRSP-MER/2011-IV. http://www.nrsp.org.pk/publications.html. Accessed 30 Jul 2016

[18] Khattak MS, Sajjad A (2015). Assessment of temperature and rainfall trends in Punjab province of Pakistan for the period 1961–2014. J Himal Earth Sci.

[19] Lanciotti RS, Calisher CH, Gubler DJ, Chang G-J, Vorndam AV (1992). Rapid detection and typing of Dengue viruses from clinical samples by using reverse trasnciptasepolymerase chain reaction. J Clin Microbiol.

[20] World Health Organization. Dengue hemorrhagic fever: diagnosis, treatment and control. Geneva: World Health Organization; 1986.

[21] Fontenille D, Failloux AB, Romi R, Takken W, Knols BGJ (2007). Should we expect Chikungunya and Dengue in Southern Europe?. Emerging Pests and Vector-Borne Diseases in Europe.

[22] Gupta E, Dar L, Kapoor G, Broor S. The changing epidemiology of *dengue* in Delhi, India. Virol J. 2006. doi:10.1186/1743-422X-3-92. 10.1186/1743-422X-3-92 PMC1636631 17083743

[23] Aziz MM, Hasan KN, Hasanat MA, Siddiqui MA, Salimullah M, Chowdhury AK (2002). Predominance of the DEN-3 genotype during the recent dengue outbreak in Bangladesh. Southeast Asian J Trop Med Public Health.

[24] Aghaie A, John A, Sadegh C, Matthias N, Soudabeh B, Hashem KM. Frequency of dengue virus infection in blood donors in Sistan and Baluchestan province in Iran. Transfus Apher Sci. 2014. doi:10.1016/j.transci.2013.07.034. 10.1016/j.transci.2013.07.034 24332363

[25] Raheel U, Faheem M, Riaz MN, Kanwal N, Javed F, Qadri I (2011). Dengue fever in the Indian subcontinent: an overview. J Infect Dev Ctries.

[26] Warraich H, Zaidi AK, Patel K. Floods in Pakistan: a public health crises. Bulletin of the World Health Organization; 2011. doi:10.2471/BLT.10.083386. 10.2471/BLT.10.083386 PMC3044252 21379421

[27] Joshi V, Mouriya DT, Sharma RC (2002). Persistence of Dengue-3 virus through transovarial transmission passage in successive generations of aedes aegypti mosquitoes. Am J Trop Med Hyg.

[28] Sharmin S, Viennet E, Glass K, Harley D. The emergence of dengue in Bangladesh: epidemiology, challenges and future disease risk. Trans R Soc Trop Med Hyg. 2015. doi:10.1093/trstmh/trv067. 10.1093/trstmh/trv067 26333430

[29] Tang JW, Khanani MR, Zubairi AM, Lam WY, Lai F, Hashmi K, et al. A wide spectrum of dengue IgM and PCR positivity post-onset of illness found in a large dengue 3 outbreak in Pakistan. J Med Virol. 2008. doi:10.1002/jmv.21290. 10.1002/jmv.21290 19040287

[30] Humayoun, MA, Waseem T, Jawa AA, Hashimi MS, Akram J. Multiple dengue serotypes and high frequency of dengue hemorrhagic fever at two tertiary care hospitals in Lahore during the 2008 dengue virus outbreak in Punjab, Pakistan. Int J Infect Dis. 2010. doi:10.1016/j.ijid.2009.10.008. 10.1016/j.ijid.2009.10.008 20171916

[31] Araújo FM, Nogueira RM, de Araújo JM, Ramalho IL, Roriz ML, de Melo ME, Coelho IC (2006). Concurrent infection with dengue virus type-2 and DENV-3 in a patient from Ceara, Brazil. Mem Inst Oswaldo Cruz.

[32] Vinodkumar CS, Kalapannavar NK, Basavarajappa KG, Sanjay D, Gowli C, Nadig NG, Prasad BS. Episode of coexisting infections with multiple dengue virus serotypes in central Karnataka, India. J Infect Public Health. 2013. doi:10.1016/j.jiph.2013.01.004. 10.1016/j.jiph.2013.01.004 23806706

[33] Christie B (2000). Doctors revise Declaration of Helsinki. BMJ.

